# *In Situ* 3D Segmentation of Individual Plant Leaves Using a RGB-D Camera for Agricultural Automation

**DOI:** 10.3390/s150820463

**Published:** 2015-08-19

**Authors:** Chunlei Xia, Longtan Wang, Bu-Keun Chung, Jang-Myung Lee

**Affiliations:** 1 The Research Center for Coastal Environmental Engineering and Technology of Shandong Province, Yantai Institute of Coastal Zone Research, Chinese Academy of Sciences, Yantai 264003, China; E-Mail: c.xia2009@gmail.com; 2 School of Electrical Engineering, Pusan National University, Busan 609-735, Korea; E-Mail: longtan7379@pusan.ac.kr; 3 Division of Plant Environment, Gyeongsangnam-Do Agricultural Research and Extension Services, Jinju 660-985, Korea; E-Mail: bkchung@korea.kr

**Keywords:** plant monitoring, occlusions, leaf detection, mean shift, center of divergence, automatic initialization

## Abstract

In this paper, we present a challenging task of 3D segmentation of individual plant leaves from occlusions in the complicated natural scene. Depth data of plant leaves is introduced to improve the robustness of plant leaf segmentation. The low cost RGB-D camera is utilized to capture depth and color image in fields. Mean shift clustering is applied to segment plant leaves in depth image. Plant leaves are extracted from the natural background by examining vegetation of the candidate segments produced by mean shift. Subsequently, individual leaves are segmented from occlusions by active contour models. Automatic initialization of the active contour models is implemented by calculating the center of divergence from the gradient vector field of depth image. The proposed segmentation scheme is tested through experiments under greenhouse conditions. The overall segmentation rate is 87.97% while segmentation rates for single and occluded leaves are 92.10% and 86.67%, respectively. Approximately half of the experimental results show segmentation rates of individual leaves higher than 90%. Nevertheless, the proposed method is able to segment individual leaves from heavy occlusions.

## 1. Introduction

Precision agriculture is a synthesis technology that enhancing crop production with minimal energy costs and environmental pollution [1,2]. Recently, precision agriculture has been rapidly developed by introducing advanced technologies, such as intelligent sensors and robotics. The productivity of the conventional farming, which the crop cultivation and management manually conducted by farmers, is significantly improved by using intelligent machines. Numbers of agricultural robotics based on visual guidance are developed for automatic agricultural operations, such as micro-dosing, plant de-leafing, weed control and pest management [3,4,5,6]. Due to the success of computer vision techniques, imaging sensors have become the most common sensing devices in agricultural automation systems for collecting information of plants.

Image analysis of plant leaves is one of the essential tasks for agricultural automation since a plant leaf contains abundant information of plants. In particular, automatic detection of individual leaves is a fundamental task for achieving precision operations in agricultural practices. Segmentation of individual leaves is a challenging task due to plant leaves showing significantly varying poses and complex shapes in natural conditions. Plant leaf segmentation has been extensively studied in the last decades [7]. Numerous sensing techniques are presented to acquire plant information, such as image camera, infrared camera, spectral camera, *etc*. In the initial stage, plant leaf segmentation is mainly conducted by using 2D segmentation approaches. Genetic algorithms are introduced to extract individual leaves from canopy images [8]. Watershed based leaf segmentation is reported in [9], which efficiently extracts plant leaves from the images taken from tomato fields. Neural network is also demonstrated high performance in detecting vegetation pixels and extract leaves from the ground [10].

These works are successful in extracting leaves from the natural background. However, these methods are less able to identify leaves from occlusions and might be sensitive to illuminations changes. Occlusions frequently occur in plant images. In order to deal with detecting occluded plant leaves deformable models containing *a priori* knowledge on leaf shapes are presented to segment the individual leaves from the complicated background. A parametric deformable template is developed to accumulate information on weed leaves on the basis of the tips of leaves [11]. Lately, the active shape model (ASM) is utilized to detect occluded leaves in field conditions with *a priori* knowledge. A modified active shape model is presented to detect occluded and damaged pepper leaves in greenhouses [12]. The active shape model shows high accuracy in identification of weed species [13].

Recently, 3D segmentation of plant leaves has attracted attention by the scientists and engineers due to the rapidly developed imaging systems and highly improved computational performance of computers. Various imaging systems are developed to obtain 3D plant images, such as stereo camera and laser scanners. Three-dimensional reconstruction from multiple views (e.g., stereo vision) is one of the successful techniques to obtain accurate 3D information of objects. Quan *et al.* developed an image-based plant modeling system that can reconstruct the 3D model of a plant from a set of images around the plant [14]. This method measures point cloud data of plants from more than 30 images taken from different angles of views. A graph based segmentation scheme integrating 3D and 2D information is developed to segment leaves and to reconstruct 3D structure of plant leaves by fitting leaf models. Another 3D leaf segmentation system is devised based on multiple views camera and classification of plant species is implemented by using 3D leaf images [15]. A mesh processing based precise 3D measurement of plant leaves and stems are proposed based on the multiple views of plants [16]. And the plant species could be identified by the 3D leaf images. Since the collection of multiple view images of plants should be conducted in controlled conditions, such as in a laboratory, this approach is not yet available for agricultural applications.

High precision 3D data of plants could also be obtained by using 3D Laser scanners. Detection of individual plant in crop fields using LIDAR sensor is developed for agricultural robots navigation [17]. Plant phenotyping is implemented based on the point cloud obtained by a laser scanner, and the plant organs, such as leaves and stems, are classified by using the laser-scanned data [18]. A 3D dynamic measurement system is developed based on the LIDAR sensor and the structure of a fruit tree and the leaf area are estimated [19].

Furthermore, a time of flight (ToF) camera is developed for measuring accurate 3D information of objects and has been applied to 3D analysis of plants. Plant phenotyping has been developed by using a ToF camera [20]. An automatic leaf grasping by manipulator is presented by measuring individual leaves using a ToF camera [21]. In this work, individual plant leaves are segmented by applying graph-based segmentation and fitting quadratic leaf models to depth data. Moreover, the suitability of using ToF cameras for agricultural applications is evaluated by comparing with stereo cameras under the indoor and outdoor conditions [22,23]. The ToF cameras show comparable accuracy with stereo vision.

Although stereo vision and laser scanner can provide high precision 3D data of plants, currently, they are not affordable for agricultural practices due to their extremely high prices. A ToF camera is an alternative solution for acquiring real-time depth images. However, most ToF cameras provide low resolution of depth image (e.g., 204 × 204 pixels for PMD^®^ CamCube). Low cost RGB-D cameras have recently been developed for 3D imaging with better image resolution than a ToF camera (e.g., 640 × 480 pixels). RGB-D cameras have been utilized in numerous applications in 3D reconstruction, object recognition and remote control [24,25,26]. RGB-D cameras have been evaluated for plant phenotyping by comparing with the high precision laser scanners. The low-cost RGB-D imaging devices are shown impressive reliability for 3D plant phenotyping and the possibility to automated agricultural applications is demonstrated [27]. The 3D structure of plants is reconstructed by using depth data [28]. Moreover, an indoor test of 3D plant phenotyping using RGB-D camera is conducted in [29]. The leaf segmentation using depth is presented and the feasibility of plant monitoring based on RGB-D camera is proved.

In the previous works, the RGB-D camera showed high performance in plant image analysis. The RGB-D camera could produce real-time depth data which is less computational cost for 3D mapping. The RGB-D camera is extremely economic compared with laser and stereo cameras. However, plant leaf analysis using a RGB-D camera in a natural scene has not been extensively studied. We focus on plant leaf segmentation for agricultural automation in practice. This work presents a 3D leaf segmentation scheme under natural conditions and an accurate measurement of individual leaves from heavy occlusion. The mean shift segmentation is introduced to segment leaves from the complicated background in green house fields. An automatic initialization of active contour model (ACM) is implemented by calculating the center of divergence (CoD). Occluded individual leaves are segmented by using the ACM. Performance of the proposed segmentation scheme is verified through field experiments in a green house.

## 2. Materials

**Figure 1:**
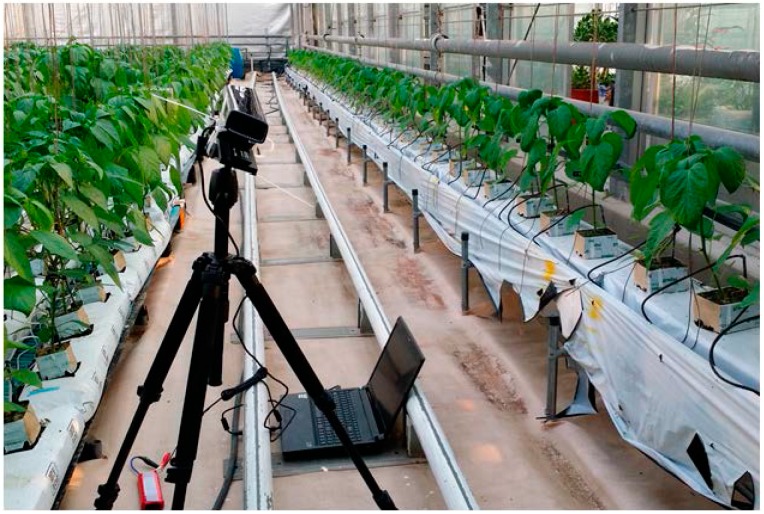
Structure of plant monitoring using Kinect camera in the green house.

A Kinect RGB-D camera developed by Microsoft^®^ Company (Redmond, WA, USA) is adopted to capture color and depth images of plants. The structure of plant monitoring facility is presented in Figure 1. A Kinect camera is installed on a tripod and connected to a laptop computer for capturing images. The Kinect camera is powered by a 12V Li-On battery. The Kinect camera could produce 1280 × 960 pixels color images and 640 × 480 pixels depth images. To align the color image with depth image the resolutions of RGB and depth images from Kinect are set to 640 × 480 pixels. The Kinect camera and tripod is positioned 100 cm away from the plants in a horizontal direction and the Kinect camera is placed at 130 cm height with 30° downward angle to capture the optimized view of plants (Figure 1).

## 3. Segmentation of Plant Leaves

### 3.1. Overall Procedure of Plant Leaf Segmentation

**Figure 2:**
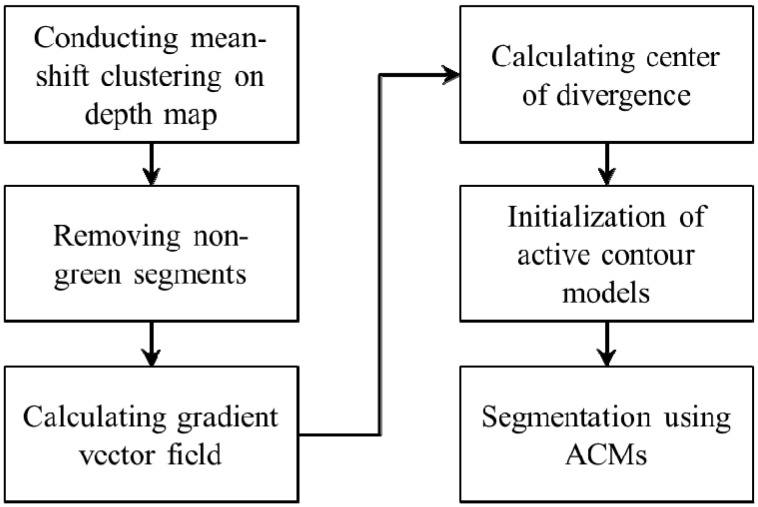
Flowchart of the proposed leaf segmentation scheme.

The proposed leaf segmentation scheme is conducted in mainly two steps: background removal and segmentation of individual leaves. The overall procedure of the proposed segmentation scheme is presented in Figure 2. Depth and color images of plants are captured by the Kinect camera and concurrently depth and color images are aligned for further process. Initially, mean shift clustering is conducted on the depth image to segment objects from the background. The candidate segments are examined by RGB colors. The non-green background objects are removed and only plant leaves are extracted at this step. Background images are removed from the depth and color images, respectively. Subsequently, the gradient vector field (GVF) is calculated on the filtered depth image. The center of divergence is calculated based on the gradient vector field. The automatic initialization of active contour models is implemented according to the center of divergence of the depth image. Accordingly, individual leaves are segmented from the depth image by using the active contour models.

### 3.2. Removal of Natural Background

**Figure 3:**
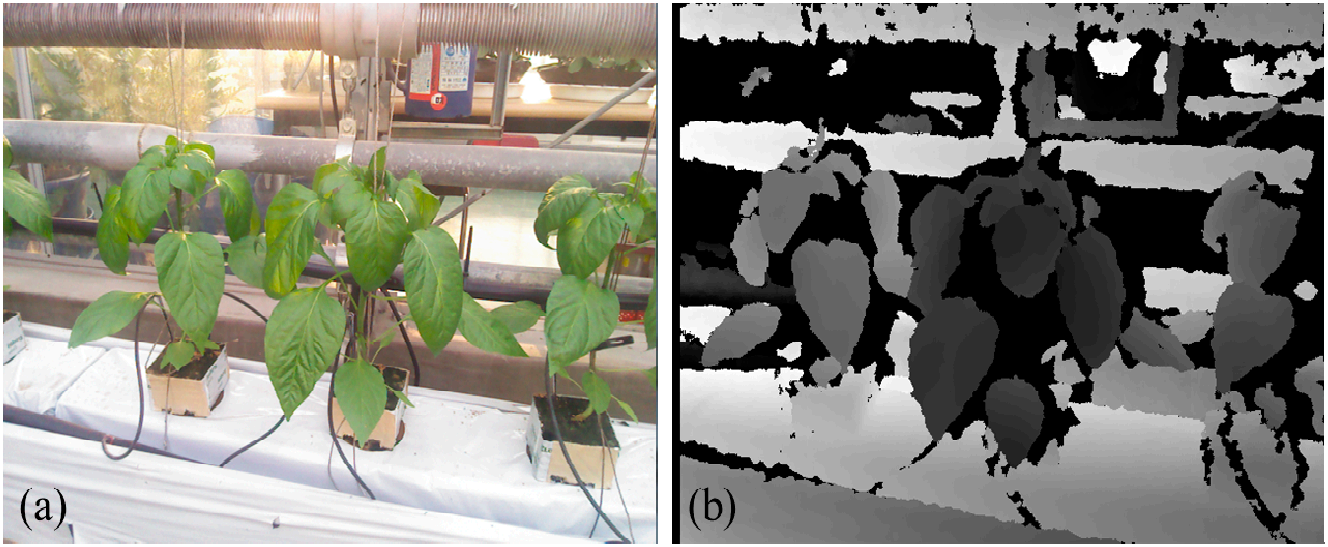
Leaf image captured by Kinect camera, (**a**) RGB image of plants and (**b**) depth image.

In plant image analysis, segmentation of plant leaves from the background in fields is a crucial and challenging task. Plant leaf segmentation should be conducted prior to the analysis of individual leaves. We introduce depth information to improve the robustness of *in situ* leaf segmentation. Plant leaves are initially extracted from the background according to the depth feature. Since depth data represent the coordinates of objects in three-dimensional space, plant leaves and background objects could be efficiently separated in terms of the discontinuity in depth. Figure 3 presents the plant images taken from green house by using Kinect camera. The RGB image and depth image of plants are obtained simultaneously (Figure 3). The illumination condition is complicated which a large area of sunlight diffusion is appeared in the top part of image (Figure 3a). Leaves are in various poses and occluded with each other. In contrast, leaves present significant difference in depth compared with the background objects (Figure 3b). The depth image of plant leaves is relatively smooth and depth noises could not represent the segmentation of leaves.

The mean shift algorithm is applied to segmented objects in the depth image. The mean shift algorithm is a robust feature-space analysis approach which has been widely applied in image segmentation and object tracking [30,31]. Mean shift is an iterative procedure that shifts each data point to the average of data points in its neighborhood by using kernel density estimation.

Given a set of *d*-dimensional points ${\left\{x_{i}\right\}}_{i=1...n}$ in Euclidian space $R^{d}$ and the mean shift vector at location $x_{i}$ is computed by

(1) $$M(x)=\frac{\sum_{i=1}^{n}x_{i}K_{W}(x_{i}-x)}{\sum_{i=1}^{n}K_{W}(x_{i}-x)}-x$$

where the kernel function

$$K_{W}(x)\;=\;{\left|W\right|}^{-1/2}K\left(W^{-1/2}x\right)$$

and x is the center of the kernel. *W* is a symmetric positive define d×d bandwidth matrix which is usually a diagonal matrix in the actual applications. In mean shift clustering, the mean shift vector M(x) calculates the direction of the maximum increase in the density and estimates the local density gradient of similar pixels. The kernel iteratively shifts to the local density peaks until convergence. The local density peaks are obtained by using Equation (2).

(2) $$y_{j+1}=\frac{\sum_{i=1}^{n}x_{i}K_{W}(y_{j}-x_{i})}{\sum_{i=1}^{n}K_{W}(y_{j}-x_{i})},j=1,2,\ldots$$

The local density peak $y_{j+1}$ is updated iteratively during the clustering process. All the points that are drawn upwards to the same peak are considered to be members of the same segment. This is the overall procedure of mean shift segmentation.

In this work, the clustering process of mean shift is conducted on the gray-level depth data to obtain the leaf areas and backgrounds. Let ${\left\{x_{i}\right\}}_{i=1...n}$ be the 2-dimensional input in the spatial-range domain of a gray-level image. Moreover, $y_{i,j}$ is the local density peak for a pixel $x_{i}$ at the *j-*th iteration. The procedure of mean shift segmentation on depth data is described in the following steps:

1. Initializing a searching window in spatial domain with radius $s_{p}$ and range domain window with radius $s_{r}$ and set iteration step j=1 and local density peak $y_{i,1}=x_{i}$.
2. The shift vector of searching windows is obtained by examining the depth difference between each pixel and the center pixel in the spatial window and range window.
3. Computing the mean depth value of all the pixels within the spatial windows and updating $y_{i,j+1}$ with the mean depth.
4. Pixels in the spatial window which showing depth difference smaller than radius $s_{r}$ of range window are grouped into the clusters ${\left\{C_{p}\right\}}_{p=1...m}$. The above steps are repeated until the shift value is small enough that indicate the mean shift clustering is converged. Assign $z_{i}=y_{i,c}$.
5. Finally, each pixel $x_{i}$ is grouped into the segments by assigning $L_{i}=\{p|z_{i}\in C_{p}\}$ (refer to [30] for detailed algorithms).

**Figure 4:**
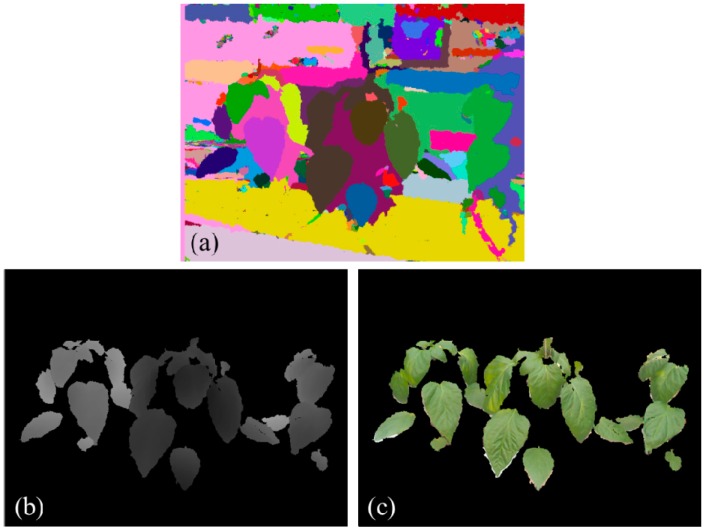
Leaf segmentation using mean shift, (**a**) mean shift segmentation results; (**b**) segmentation results presented in depth image and (**c**) segmented leaves in RGB image.

Consequently, the depth image is segmented into numbers of sub-areas when the convergence of mean shift is reached. As presented in Figure 4a, segments are represented by different colors. The leaf images are determined from the segmentation results by examining the RGB color of green vegetation as shown in Figure 4. Non-green background objects are removed in this process. Plant images are segmented into numerous sub-images of leaves and only segmented leaves are extracted in depth and color images (Figure 4b,c).

A standard mean shift clustering implemented by the open source computer vision library (OpenCV 2.4) is adopted. The segmentation of mean shift is optimized by tuning the spatial window radius $s_{p}$ and the feature window radius $s_{r}$ according to depth difference. $s_{p}$ determines the size of segments and $s_{r}$ is related to the depth difference among objects. In this work, the background segmentation is not sensitive to these two parameters because the leaves and the background present significant difference in depth. The values of $s_{p}$ and $s_{r}$ are chosen as 12 and 6 in our experiments. The RGB thresholds for eliminating backgrounds are determined from the pre-tests on the field images.

### 3.3. Segmentation of Individual Leaves from Occlusions

In the background removal process, single leaves could be separated from the background while occluded leaves are difficult to be segmented by the mean shift because of the insignificant depth difference between the occluded leaves. A sophisticated method should be presented to accurately extract the individual leaves from occlusions. Considering the flexible shape of leaves and varying poses, a flexible contour model, or active contour model, could efficiently fit the accurate boundaries of leaves [32]. The active contour model based on gradient vector field is utilized to segment occluded individual leaves in depth images [33] since most occluded leaves show small depth difference on the boundaries between two leaves. The boundaries of occluded leaves could be identified using the active contour model by fitting to the local boundary feature in depth image.

Automatic initialization of the active contour model is an essential step to obtain the accurate segmentation of individual leaves. A proper number of models should be initialized at the optimized positions in the plant image. A large number of hypothesized models could not only increase the computational cost and also lead to over-segmentation of leaves. In this work, we calculate the center of divergence for initializing the active contour models [34]. The CoD corresponds to the local maxima of the external energy field which is calculated from the point from the GVF vectors of all the neighboring pixels.

The GVF vectors of pixel are calculated from the four adjacent pixels: p(i,j), p(i+1,j), p(i,j+1) and p(i+1,j+1) where *i* and *j* are the coordinates of pixels in the image. The GVF vector of pixel p(i,j) is calculated as v(i,j)=(x(i,j),y(i,j)). Subsequently, a sign function is introduced to represent the direction of v(i,j):

(3) $$sign(x)=\{\begin{array}{c} 1,\;x\;>\;0\\ 0,\;x\;=\;0\\ -1,\;x\;<\;0 \end{array}$$

Accordingly, the potential scattering point set $P_{s}$ is given as:

(4) $$\begin{array}{l} P_{sx}=\{p(i,j)|x(i,j)\leq x(i+1,j)\;and\;\\ \;abs(sign(x(i,j))\;+\;sign(x(i+1,j)))\leq 1\} \end{array}$$

(5) $$\begin{array}{l} P_{sy}=\{p(i,j)|y(i,j)\leq y(i,j+1)\;and\;\\ \;abs(sign(y(i,j))\;+\;sign(y(i+1,j)))\leq 1\} \end{array}$$

(6) $$P_{s}=P_{sx}\cap P_{sy}.$$

and the center of divergence is determined by applying a given threshold. If the distance of any two points in $P_{s}$ is larger than the threshold, one of the two points is removed.

**Figure 5:**
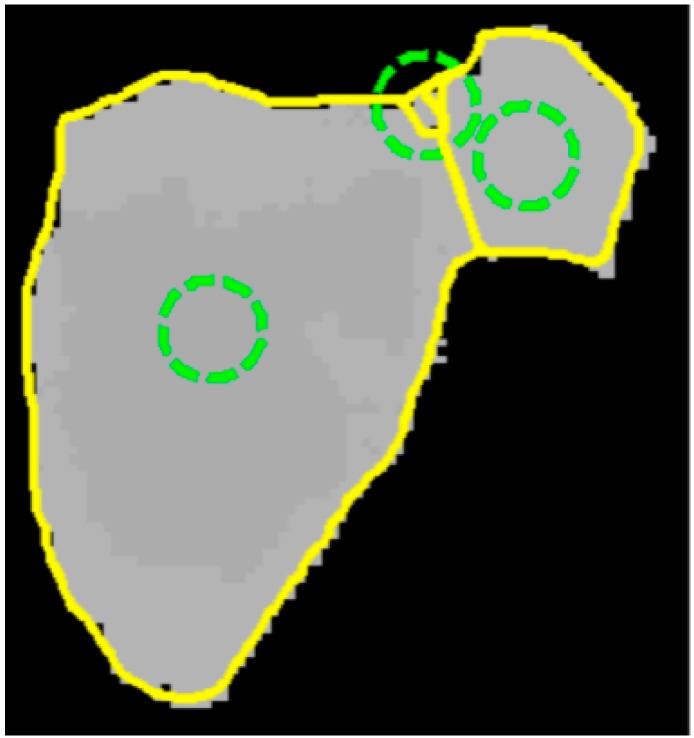
Leaf segmentation using center of divergence (CoD) and active contour model (ACM) (Green circles are initialized models and yellow contours are segmentation results).

According to the center of divergence, the contour models are initialized to segment the leaves from occlusions. The initialized models are drawn in green circles while the boundaries of segmented of individual leaves are represented in yellow lines (Figure 5).

## 4. Results

**Figure 6:**
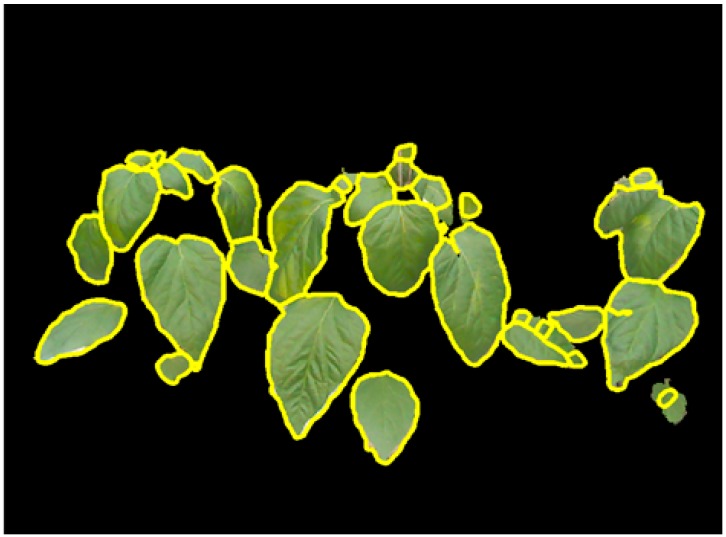
Segmentation of individual leaves (yellow contours represent boundaries of individual leaves).

Experiments of 3D leaf segmentation are carried out on the plant images captured under greenhouse conditions. In total, 37 field images of paprika plants are tested by the proposed segmentation scheme. These images contain 474 target leaves that 24.05% (114 leaves) of them are single leaves while the rest 75.95% (360 leaves) of leaves are with occlusions (Table 1). Initially, plant leaves are segmented from the background by using mean shift segmentation and color filtering using RGB colors. The results of background segmentation show that leaves are efficiently extracted from the complicated background in green house (Figure 4c). Although the illumination condition is complex in field conditions, such as shadows and reflection on leaf surfaces, the leaves could be accurately segmented from the natural scene according to the depth image and color values (Figure 3a). Figure 6 presents segmented individual leaves from plant images in Figure 3. Segmented individual leaves are drawn with yellow contours. Individual leaves showing irregular shapes and complicated poses are accurately segmented although the leaves are clustered and occluded with each other.

The performance of 3D individual leaf segmentation is presented in Table 1. The segmentation performance of individual leaves is evaluated by segmentation rate, failure detection, unseparated occlusions and over-segmented leaves. Segmentation rate indicates the proportion of accurately identified individual leaves from the total amount of individual leaves. Failure detection is the individual leaves which are unable to be detected during the background removal process. While unseparated occlusions represent the occluded leaves which are not correctly separated by the proposed method, such as the top right leaves in Figure 6. Over-segmentation is the individual leaves which is incorrectly segmented into several partial leaf images. An example of over-segmentation is shown in the middle right of Figure 6. The leaf segmentation is evaluated by examining the identified individual leaves by the proposed method with manually labeled individual leaves. Up to 92.10% of single individual leaves are correctly identified from the plant images. Segmentation rate of occluded leaves is 87.97% while the overall segmentation rate is 86.67%. The segmentation rate is remarkable: such a precise measurement of individual leaves from occlusions is challenging since leaves are showing rather complicated poses. The segmentation performance of the proposed method is remarkable that only nine leaves were failed to be detected among the overall target leaves (Table 1). The failure detection rates for single leaves and occluded leaves are 5.26% and 0.83%, respectively. Single leaves show higher failure detection rate since single leaves contain numbers of small-sized young leaves which are difficult to be measured using the Kinect camera. The experimental results show that the incorrect segmentation of individual leaves is mainly caused by over-segmentation. Especially for occluded leaves, the over-segmentation rate is 8.33% while the over-segmentation rate for single leaves is 5.26%. It is impressive that only 3.33% of the occluded leaves are unable to be separated by the proposed segmentation scheme.

**Table 1:** Segmentation performance for single and occluded individual leaves.

	Proportion of Leaves	Segmentation Rate	Failure Detection	Unseparated Occlusion	Over-Segmentation
Single leaves	24.05% (114)	92.10% (105)	5.26% (6)	N/A	5.26% (6)
Occluded leaves	75.95% (360)	86.67% (312)	0.83% (3)	3.33% (12)	8.33% (30)
Overall	100% (474)	87.97% (417)	1.90% (9)	2.53% (12)	7.59% (36)

**Figure 7:**
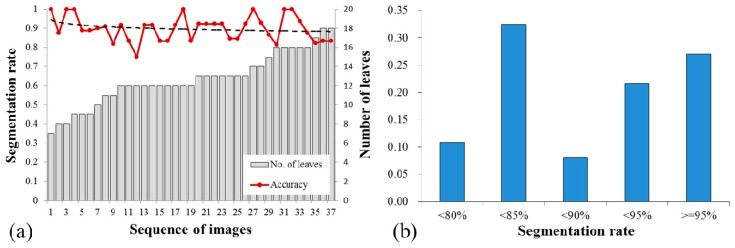
Analysis of segmentation rates for each plant images, (**a**) the trend of segmentation rates and (**b**) distribution of segmentation rates.

The segmentation performance for each plant images is investigated in Figure 7. The segmentation rates vary in the range of 75%–100% with standard derivation 8.02%. While the number of target leaves in each plant image is varied from 7 to 18. The trend line of the segmentation rate is plotted in a black dash line. Although the segmentation rate shows a slightly decreasing trend with the increasing number of leaves, the segmentation performance is reliable and high segmentation rates (>80%) are obtained for the images containing high density of leaves (>14) (Figure 7a). The distribution of segmentation rate is presented in Figure 7b and approximately 90% of the experimental results have segmentation rates of over 80%. Proportions of the segmentation rates of 80%–85%, 85%–90%, 90%–95% and ≥95% are 0.32, 0.08, 0.22 and 0.27. It is notable that approximately half of the segmentation rates are higher than 90% and 27% of results show segmentation rates more than 95%.

## 5. Discussion

In this work, automatic segmentation of individual leaves is implemented in a green house. The proposed 3D leaf segmentation scheme could accurately extract individual plant leaves from field images. Generally, *in situ* segmentation of plant leaves encounters the following issues: image noises from the complicated natural background, varying illumination conditions, flexible leaf shapes and occlusions of leaves. Varying illumination and color distortion frequently appeared in the field images. The ground is always confusing with coloured leaves under field conditions, which could lead to inaccurate leaf segmentation in color images. Numerous studies have been proposed to filter the vegetation pixels from the natural background [35,36,37]. Our experimental results prove that introducing depth information could significantly improve the efficiency and stability of plant leaf segmentation under natural conditions. In our experiments, most leaves containing shadow and reflection are correctly measured. Since the intensity of sunlight in the late afternoon is relatively low, it could not effectively interfere with the measurement of the Kinect camera in green house conditions. The experimental results suggest that the measurement of plant leaves using RGB-D camera could be conducted directly in fields under low intensity of light conditions (e.g., early morning or late afternoon) or with additional facilities reducing the intensity of light under all natural conditions. The RGB-D camera is also able to measure the 3D information of plants at night since infrared light is utilized to depth measurement. However, additional lighting is required to acquire color images of plants at night.

The proposed leaf segmentation using depth data is robust to background noises and complex illumination conditions (e.g., shadows). Because plant leaves and the background (e.g., ground) have large difference in depth in the camera coordinates. Mean shift clustering could efficiently extract the leaves from background according to their depth. In this process, accurate segmentation of individual leaves could be obtained for those single leaves or occluded individual leaves which showing significant difference in depth to adjacent leaves. However, occluded leaves are commonly attached close to each other and showing small differences in depth which are difficult to separate using the clustering algorithm.

**Figure 8:**
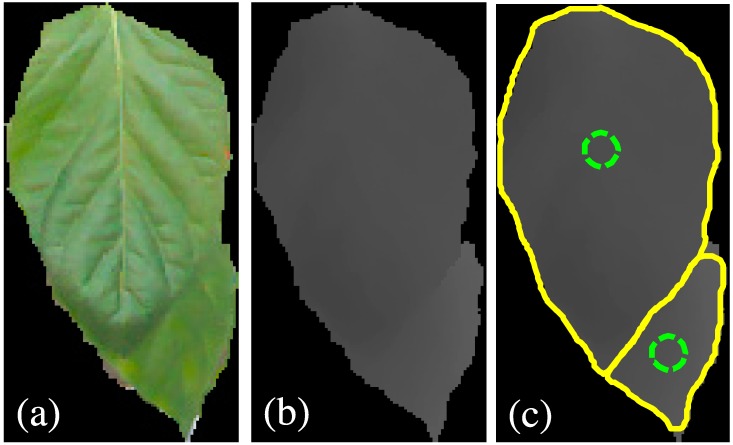
Segmentation of individual leaves from heavy occlusions. (**a**,**b**) are the segmented leaves of color and depth image, and (**c**) is the segmentation results in which green circles are initialized models and segmented leaves are presented in yellow contours.

The challenge of this work is to segment leaves from the heavy occlusion in natural conditions. As presented in Figure 8a, leaf boundary of the occluded part is difficult to detect even by human eyes and the leaf veins produce rather serious noise in color images. In contrast, the boundary between leaves is observed and leaf veins disappeared in the depth image (Figure 8b). The depth image could provide boundary feature for the segmentation of individual leaves from heavy occlusions. The active contour model could exactly fit the leaf boundary the depth image. The CoD method could properly calculate the local minima in the gradient vector field of depth image for initialing the contour models. Initialization positions of the contour models are plotted in the green circles in Figure 8c. The segmentation performance of heavy occlusions is remarkable since more than 2/3 of the surface of the underneath leaf is invisible and the entire shape of the whole image is confusing to a regular leaf. Nevertheless, the experimental results show that the CoD procedures show a proper number of contour models, and over-segmentation is reduced in this case.

**Figure 9:**
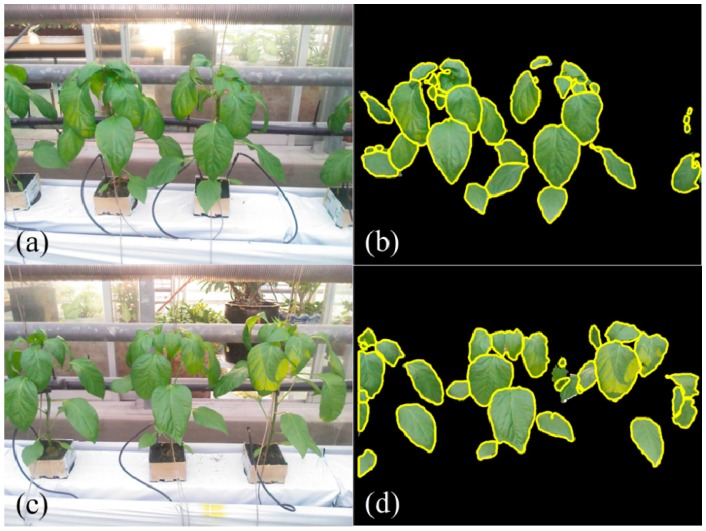
Plant images and segmentation results, (**a**,**c**) are field images and (**b**,**d**) are their corresponding segmentations.

Since this work deals with plant leaves in complex poses in fields, many of the leaves are in irregular shapes. It is difficult to model the leaf shapes by using geometric models or statistical models, such as parametric models and active shape models [11,12]. The deformable contour model has the ability to fit the leaf boundaries of irregular shapes according to the discontinued depth between leaves. Various shapes of leaves are exactly segmented by the active contour models, including twisted leaves, side-view leaves and leaves in irregular shapes. In particular, it is difficult to measure the accurate depth information of leaves in irregular poses, such as wilted, rolled or twisted leaves. Examples of field images and their segmentation results are presented in Figure 9. Clustered individual leaves are accurately segmented and leaves with shadow are also correctly identified (Figure 9d). In our tests, incorrect leaf segmentations are usually caused by measurement error of depth. Although most irregular leaves are correctly segmented by the proposed method, a small proportion of them with severely distorted poses are the main reason of inaccurate segmentations. For instance, wrinkles on leaf surface produce a discontinuous depth that results in over-segmentation of leaves. In this case, the plant leaf is recognized as several small pieces of partial leaves. As presented in Figure 9c,d, the right side leaf is divided into small parts since the leaf surface is rolled and depth of the leaf is discontinuity. Side-view leaves in Figure 9a,c are lost in the segmentation results due to the hardware limitation of the Kinect camera. Reflection on leaf surface produces noises to depth data and lead to incorrect segmentation as shown in Figure 9d.

Moreover, the proposed method is difficult to segment the occluded leaves showing tiny difference in depth. As presented in the top right leaves in Figure 6, the two leaves show smooth depth. In this case, the boundary of occluded part could not be detected by calculating gradient vector field. Similar segmentation results appear in the middle of Figure 9d where the two small leaves are attached together. The proposed segmentation scheme is developed based on the depth feature of leaves. Therefore, the proposed method could be effective for segmenting occluded leaves showing significant difference in depth.

Another advantage of the proposed scheme is that it is insensitive to parameter setting and all of the experiments are conducted using the constant parameters for each segmentation steps. The parameter setting is important to the automation systems and our results indicate that the proposed method could automatically observe the leaf status without human interference.

Due to the physical limitation of RGB-D camera inaccurate measurement is occurred in the depth data. The precision of depth measurement of the Kinect camera is limited that implies the detailed depth information of objects could be lost and small-sized objects might not be detected by the Kinect camera. Noises frequently occur in the measurement data, the boundaries of leaves are not smooth and leaf tips might not be measured in the depth image. Side-view leaves might not be correctly measured if they show large angles (>60°) to the imaging plane because these leaves present small visible area and large slope in depth in the camera view (Figure 9). In this work, the working distance of the Kinect camera is set to 100 cm so that the depth of small objects could be inaccurate in the tests. Therefore, small leaves are not taken into account in the experiments. Moreover, plant stems are almost impossible to capture with the Kinect camera due to their thin body. The measurement of stems is out of the range of this work; it could be considered in the further studies. Acquiring more detailed 3D information of plants may need additional facilities, such as utilizing high performance graphics cards and computers, but this solution is not economic for agricultural applications and it is not necessary for agricultural operations. Precision of depth provided by the low-cost Kinect cameras could satisfy the requirement of plant monitoring or robot operations. Agricultural automation using a RGB-D camera has high price-performance ratio comparing with the stereo vision and laser scanners. Furthermore, the computational time of background removal is 2289.7 ms and segmentation of individual leaves costs 150.4 ms on average on a personal computer (Intel^®^ G860 CPU 3 GHz and 8G RAM). The computational time of the proposed method is acceptable for actual applications since real-time processing is not necessary for plant monitoring or agricultural operations by robots. Therefore, the proposed 3D leaf segmentation scheme based on RGB-D camera for agricultural automation is practical and it could be extended to large scale deployment in agricultural applications, such as sensing network for plant monitoring.

We demonstrate the feasibility of *in situ* plant leaf measurement using RGB-D camera. Individual plant leaves are successfully extracted from the natural scene. The experimental results prove the proposed method has great potential in the agricultural applications. Leaf segmentation is tested on the paprika plants and the proposed method could also be applied to many other species of plants. However, the development of leaf monitoring presented in this work is still in its initial stage. Segmentation of leaves is conducted on depth and color images separately. We tried several schemes to modify the mean shift segmentation by integrating depth and color to enhance the stability of the segmentation. For example, depth image is integrated to the color image in RGB or Lab color space by multiplying a weight value and the mean shift is accordingly conducted on the new image. Unfortunately, the combination of depth and color image is failed to improve the stability of segmentation. More tests should be carried out to enhance the segmentation efficiency in the further works. The influence of illumination conditions on the leaf segmentation should also be investigated in depth. Furthermore, 3D posture of leaves could be calculated based on the 3D segmentation results of this work. The 3D posture analysis of plant leaves should be studied in the further works to obtain the accurate plant status. Nevertheless, measuring actual size of leaves using RGB-D camera could be studied in the next step; this is not addressed in this work. The precision of depth measurement could be improved by adopting new Kinect v2, which is based on the ToF technology.

## 6. Conclusions

In this paper, a 3D segmentation of individual plant leaves is presented by using a low cost RGB-D camera. Plant leaf segmentation from a natural background is implemented based on mean shift clustering. Individual leaves with occlusions are segmented accurately by using an active contour model. The automatic initialization of contour models is developed by calculating the center of divergence from the gradient vector flow in depth image. Remarkable segmentation rates for both single and occluded leaves are obtained through the field experiments. Plant leaves in heavy occlusion could be correctly identified. The feasibility of *in situ* plant leaf measurement using RGB-D camera is demonstrated through field tests. The proposed segmentation scheme could be applied to many related works in agricultural automation, such as de-leafing, plant inspection and pest management. This work could also be adapted to harvesting agricultural products (e.g., fruits) by greenhouse mobile robots in the future.
